# Histidine limitation alters plant development and influences the TOR network

**DOI:** 10.1093/jxb/erae479

**Published:** 2024-12-17

**Authors:** Amandine Guérin, Caroline Levasseur, Aline Herger, Dominik Renggli, Alexandros Georgios Sotiropoulos, Gabor Kadler, Xiaoyu Hou, Myriam Schaufelberger, Christian Meyer, Thomas Wicker, Laurent Bigler, Christoph Ringli

**Affiliations:** Department of Plant and Microbial Biology, University of Zurich, and Zurich-Basel Plant Science Center, Zurich, Switzerland; Department of Plant and Microbial Biology, University of Zurich, and Zurich-Basel Plant Science Center, Zurich, Switzerland; Department of Plant and Microbial Biology, University of Zurich, and Zurich-Basel Plant Science Center, Zurich, Switzerland; University of Zurich, Department of Chemistry, Zurich, Switzerland; Department of Plant and Microbial Biology, University of Zurich, and Zurich-Basel Plant Science Center, Zurich, Switzerland; Department of Plant and Microbial Biology, University of Zurich, and Zurich-Basel Plant Science Center, Zurich, Switzerland; Department of Plant and Microbial Biology, University of Zurich, and Zurich-Basel Plant Science Center, Zurich, Switzerland; Department of Plant and Microbial Biology, University of Zurich, and Zurich-Basel Plant Science Center, Zurich, Switzerland; Institut Jean-Pierre Bourgin (IJPB), INRAe, AgroParisTech, Université Paris-Saclay, Versailles, France; Department of Plant and Microbial Biology, University of Zurich, and Zurich-Basel Plant Science Center, Zurich, Switzerland; University of Zurich, Department of Chemistry, Zurich, Switzerland; Department of Plant and Microbial Biology, University of Zurich, and Zurich-Basel Plant Science Center, Zurich, Switzerland; University of Antwerp, Belgium

**Keywords:** Amino acid metabolism, Arabidopsis, HISN2, histidine deficiency, LRX1, LRX1ΔE14, root growth, root hair, *sune82*, TOR

## Abstract

Plant growth depends on growth regulators, nutrient availability, and amino acid levels, all of which influence cell wall formation and cell expansion. Cell wall integrity and structures are surveyed and modified by a complex array of cell wall integrity sensors, including leucine-rich repeat (LRR)-extensins (LRXs) that bind RALF (rapid alkalinization factor) peptides with high affinity and help to compact cell walls. Expressing the Arabidopsis root hair-specific LRX1 without the extensin domain, which anchors the protein to the cell wall (*LRX1ΔE14*), has a negative effect on root hair development. The mechanism of this negative effect was investigated by a suppressor screen, which led to the identification of a *sune* (*suppressor of dominant-negative LRX1ΔE14*) mutant collection. The *sune82* mutant was identified as an allele of *HISN2*, which encodes an enzyme essential for histidine biosynthesis. This mutation leads to reduced accumulation of histidine and an increase in several amino acids, which appears to have an effect on the TOR (target of rapamycin) network, a major controller of eukaryotic cell growth. It also represents an excellent tool to study the effects of reduced histidine levels on plant development, as it is a rare example of a viable partial loss-of-function allele in an essential biosynthetic pathway.

## Teaser

Partial loss of function of *HISN2* in *sune82* results in a significant reduction in histidine content, which subsequently alters plant development and the TOR network.

## Introduction

Growth and development of any given organism depend on the availability of nutrients and essential organic molecules such as vitamins or amino acids used for protein biosynthesis. Unlike many animals including humans, plants are able to synthesize all amino acids (Trovato *et al.*, 2021). The homeostasis of the different amino acids is essential for and depends on the growth and metabolic activity of the organism, since amino acids are not only used for protein translation, but they also serve as signals, precursors for secondary metabolites and, in the case of BCAAs (branched chain amino acids) and Lys, as a carbon source for respiration (Heinemann and Hildebrandt, 2021). The biosynthesis of amino acids is tightly controlled, and their availability in turn influences cellular activities. The TOR (target of rapamycin) network is a key regulator of cell growth in eukaryotes (Loewith, 2011; McCready *et al.*, 2020). In response to growth factors and amino acids, TOR regulates metabolic and translational activities, as well as the dynamics of cellular processes such as cytoskeletal organization (Dobrenel *et al.*, 2016a; González and Hall, 2017; Cao *et al.*, 2019; Lutt and Brunkard, 2022). Central to this pathway is the TOR kinase, a phosphatidylinositol 3-kinase-related protein kinase that forms a TOR complex with interacting proteins such as RAPTOR and LST8 (Harris and Lawrence, 2003; Moreau *et al.*, 2012). Several downstream targets of the TOR kinase have been identified, one of the best characterized being the S6 kinase, which phosphorylates ribosomal protein S6 (RPS6) to influence ribosomal activities (Xiong and Sheen, 2015; Dobrenel *et al.*, 2016b). The TOR kinase can be specifically inhibited by rapamycin, which was instrumental in the identification of the TOR kinase (Chan *et al.*, 2000), but also by a new generation of inhibitors, including AZD-8055, which are valuable in modifying the TOR network (Montané and Menand, 2013). A number of proteins involved in the TOR network have been identified in various organisms, including plants, based on the observation of altered sensitivity to TOR kinase inhibitors (Leiber *et al.*, 2010; Barrada *et al.*, 2019; Cao *et al.*, 2019; Schaufelberger *et al.*, 2019; Chen *et al.*, 2023).

An important regulator that directly interacts with the TOR kinase is FERONIA (FER), a CrRLKL-type transmembrane receptor protein that plays a central role in the control of a multitude of processes, including cell wall integrity sensing, cell growth, cell–cell recognition and cell fusion during fertilization, and immune responses (Escobar-Restrepo *et al.*, 2007; Stegmann *et al.*, 2017; Feng *et al.*, 2018; Ortiz-Morea *et al.*, 2021; Song *et al.*, 2022). FER functions in conjunction with leucine-rich repeat (LRR)-extensins (LRXs), extracellular proteins that serve as binding sites for RALF (rapid alkalinization factor) peptides that help restructure the cell wall matrix by inducing pectin compaction (Dünser *et al.*, 2019; Herger *et al.*, 2020; Moussu *et al.*, 2020, 2023; Zhang *et al.*, 2020; Schoenaers *et al.*, 2024). LRX1 of Arabidopsis is expressed in root hairs, and the defect in root hair development of the *lrx1* mutant is suppressed by altering the TOR network (Baumberger *et al.*, 2001; Leiber *et al.*, 2010; Schaufelberger *et al.*, 2019). The suppressor of *lrx1*, *rol17* (*repressor of lrx1 17*), is mutated in the gene encoding isopropylmalate synthase 1 (IPMS1), an enzyme involved in Leu biosynthesis, resulting in increased Val levels (Schaufelberger *et al.*, 2019). *rol17*, and another *IPMS1* allele, *eva1*, were found to alter the TOR network. This was evidenced by a reduction in sensitivity to the TOR inhibitor AZD-8055, which results in changes in cell growth and actin dynamics (Cao *et al.*, 2019; Schaufelberger *et al.*, 2019).

LRX proteins consist of an N-terminal LRR domain fused to an extensin domain (Herger *et al.*, 2019). The extensin domain exhibits the typical characteristics of hydroxyproline-rich glycoproteins, which are structural proteins that can form intra- and intermolecular networks stabilized by covalent interactions (Mishler-Elmore *et al.*, 2021). The extensin domain of LRX1 was demonstrated to insolubilize the protein in the cell wall matrix (Ringli, 2010). This domain is essential for LRX1 function, as expression of an LRX1 variant lacking the extensin domain, termed *LRX1ΔE14* (Ringli, 2010), fails to complement the *lrx1* mutant, and induces a root hair formation defect in wild-type plants. This dominant-negative effect is phenotypically stronger than the *lrx1* mutant (Baumberger *et al.*, 2001).

In an alternative genetic approach to identify new factors influencing LRX-related processes, a suppressor screen was performed using the *LRX1ΔE14*-expressing line. This allowed for the identification of *sune* (*suppressor of dominant-negative LRX1ΔE14*) mutants, which alleviate the root hair formation defect observed in *LRX1ΔE14*. *sune82* was identified as an allele of *HISN2*, encoding an enzyme essential for His biosynthesis. *sune82* contains a missense mutation that reduces His levels to a degree that is tolerable for the plant. The *sune82* mutation also affects the sensitivity to the TOR inhibitor AZD-8055, indicating that the TOR network is impacted in this mutant. The *sune* mutant collection was then screened for other genes that are known to affect TOR activity, leading to the identification of a new *rol17* allele, *sune106*. The identification of these *sune* mutants demonstrates that interfering with the biosynthesis of different amino acids, including non-BCAAs such as His, can affect the TOR network and alter cell growth processes.

## Materials and methods

### Plant growth and propagation

The *LRX1::LRX1ΔE14* line (referred to as the *LRX1ΔE14* line) is in the Columbia (Col) background and was described previously (Ringli, 2010). *lrx1* and the *lrx1 lrx2* double mutant are described in Baumberger *et al.* (2003). Unless otherwise described, the plants were grown on 1/2 Murashige and Skoog (MS)/vitamins with 2% sucrose, 10 mg l^–1^ myo-inositol, 0.5 g l^–1^ MES pH 5.7, and 0.6% Gelrite (Duchefa) in vertical orientation at 22 °C and a 16 h light:8 h dark cycle. For propagation and crossing, seedlings were put in soil and grown under the same temperature and light regime.

For the AZD-8055 (Chemdea CD0348) treatment, lines were germinated and grown on either DMSO or 0.2/0.4/0.6 μM AZD-8055 diluted in DMSO. The AZD-8055 stock solution was diluted so that an equal volume of treatment was added to the MS medium for the different concentrations.

For the His supplementation, l-histidine (Sigma Aldrich H8000) was dissolved in water to prepare a 100 mM stock. Seeds were directly germinated on MS medium with or without 100 μM His.

For the RPS6 phosphorylation assay, seeds were germinated on 1/2 MS/vitamins with 0.3% sucrose, 0.5 g l^–1^ MES pH 5.7, and 0.5% Phytagel. Six-day-old seedlings were transferred to 1/2 MS liquid sucrose-free medium for 24 h and then either mock or sucrose treated (0.5%) for 4 h.

### Ethyl methanesulfonate mutagenesis

Ethyl methanesulfonate (EMS) mutagenesis was performed similarly to as described by Kim *et al.* (2006). Seeds from a wild-type Col plant expressing *LRX1::LRX1ΔE14* were incubated in 100 mM phosphate buffer overnight. The next day, seeds were incubated in 100 mM phosphate buffer containing 0.2% EMS for 8 h on a shaker. The M_1_ seeds were rinsed 15 times with 300 ml of water and then grown directly on soil in 240 pots, each containing 10 plants, to propagate to the M_2_ generation. Each pot represents a batch of M_2_ seeds. On average, ~20 seeds per M_2_ plant (200 seeds per batch) were screened for seedlings with a suppressed *LRX1ΔE14* root hair phenotype. Selected putative mutant M_2_ seedlings were propagated and confirmed in the M_3_ generation. Positive lines were crossed with the non-mutagenized parental *LRX1ΔE14* line and propagated to the F_2_ generation that was analyzed for segregation of the *sune* mutant phenotypes.

### Whole-genome sequencing, CAPS marker design, and targeted sequencing

Ten F_2_ seedlings from the first backcross of *sune82* with the parental *LRX1ΔE14* line exhibiting a *sune82* phenotype were selected and pooled for DNA extraction. Whole-genome sequencing of *sune82* DNA, along with the non-mutagenized *LRX1ΔE14* line, was performed at Novogene using Illumina short read technology. Raw sequence reads from the pooled *sune82* mutants were trimmed with Trimmomatic (version 0.38) with the parameters LEADING:10, TRAILING:10 SLIDINGWINDOW:5:10, MINLEN:50. Trimmed sequence reads were mapped with the bwa software (version 0.7.17-r118) to the Arabidopsis Col reference genome (TAIR version 10) using default parameters. Resulting Bam files were sorted and duplicates removed with samtools (version 1.9). New read groups were assigned to the reads with the Picard software (version:2.27.5). Sequence variants were called with the GATK software (version 4.2). The vcftools software (version 0.1.16) was used to filter the vcf files using the parameters (--max-meanDP 7 –remove-indels). The analysis revealed three SNPs (simple nucleotide polymorphisms) on chromosome 1 linked with the *sune82* mutation. Using this information, CAPS (cleaved amplified polymorphic sequence) markers were established, and co-segregation of the SNPs with the *sune82* phenotype was analyzed.

For sequencing of the *LRX1ΔE14* construct in the identified *sune* mutants, the construct was amplified using LRX1_F1 and LRX1_TermR primers (Supplementary Table S1), targeting the promoter and terminator of *LRX1*, respectively. Due to the repetitive nature and length of the extensin coding sequence, only the *LRX1ΔE14* construct was identifiable, and amplification of the endogenous *LRX1* was not possible.

For selection of plants lacking *LRX1ΔE14*, PCR to detect *LRX1ΔE14* with the primers LRX1_F1 and LRX1_TermR (see above) was conducted. To confirm the selection, seeds produced by PCR-negative plants were then grown on kanamycin, resistance to which is conferred by the *LRX1ΔE14*-containing transgene (Ringli, 2010).

The *IPMS1* gene in *sune10* and *sune65* was amplified by PCR and sequenced using different primers flanking and distributed in the coding sequence.

### CRISPR/Cas9-induced mutagenesis

The *LRX1ΔE14* parental line was transformed using a guide RNA targeting the gene of interest (Supplementary Table S1) designed using CHOPCHOP (https://chopchop.cbu.uib.no/) and inserted into the *pAGM55261* vector (Grützner *et al.*, 2021). T_1_ plants were selected based on red fluorescent protein (RFP) fluorescence, grown in soil, and the target gene was PCR-amplified and sequenced. T_1_ plants that were heterozygous or homozygous for CRISPR/Cas9 [clustered regularly interspaced palindromic repeats (CRISPR)/CRISPR-associated protein 9]-induced mutations were propagated to the next generation. RFP-negative (to select against the continuous presence of the guide RNA-containing pAGM55261) T_2_ plants were grown and again analyzed for the CRISPR/Cas9-induced mutations.

### Quantification of amino acid levels

Plants used for amino acid quantification were grown under standard conditions for 10 d. Whole seedlings were ground in liquid N_2_ and plant material was weighed and diluted with the following extraction buffer at 1:10 w/v: 80% methanol, 19% water, and 1% formic acid. The mixtures were vortexed for 5 s and placed in a sonication bath for 10 min. The mixtures were then vortexed again for 5 s and centrifuged for 5 min at 1700 *g*. The supernatant was transferred to an LC-MS vial.

For His quantification, LC was performed on a Thermo Fisher UltiMate 3000 UHPLC (Waltham, MA, USA). The UHPLC was built with a binary RS pump, an XRS open autosampler, and a temperature-controllable RS column compartment. Sample separation was performed at 30 °C using an HSS T3 Premier column (2.1×100 mm, 1.7 μm particle size) protected by the corresponding VanGuard pre-column (2.1×5 mm, 1.7 μm particle size) from Waters. The mobile phase consisted of eluent A [H_2_O+0.02% trifluoroacetic acid (TFA)] and eluent B (MeCN+0.02% TFA). The following gradient was applied at a constant flow rate of 350 μl min^–1^: (i) 0% B isocratic from 0.0 to 0.8 min; (ii) linear increase to 90% B until 2.5 min; (iii) holding 90% B until 3 min; (iv) change until 3.1 min to the starting conditions of 0% B; and (v) equilibration for 1.9 min resulting in a total run time of 5 min. Mass spectra were acquired using a Thermo Fisher Scientific Q Exactive hybrid quadrupole-Orbitrap mass spectrometer equipped with a heated ESI (electrospray ionization) source at position B and a voltage of 3.0 kV. Sheath, auxiliary, and sweep gas (N_2_) flow rates were fixed at 40, 15, and 1 (arbitrary units), respectively. The capillary temperature amounted to 275 °C, and the auxiliary gas heater temperature was 350 °C. The S-lens RF level was set to 55.0. The instrument was calibrated at a mass accuracy ≤2 ppm with a Pierce™ LTQ Velos ESI Positive Ion Calibration Solution (Thermo). Data were acquired in full scan mode between *m/z* 50 and 750, at 70 000 full width at half maximum (FWMH), with resolution at *m/z* 200, a maximum IT of 247 ms, and an AGC target of 3e6. Xcalibur 4.1 and TraceFinder 4.1 softwares (Thermo Fisher Scientific) were employed for data acquisition, peak area integration (extracted ion chromatograms with 3 ppm MS-peak width), and quantitation.

Free amino acids were derivatizated based on the work by Cohen (2003) and the recommendations from the supplier. A mixture of 5 μl and 10 μl samples, 5 μl of 500 mM MSK-CAA-A2 (Cambridge Isotopes Laboratories) internal standard mixture, 30 μl and 25 μl of borate buffer, and 10 μl of AQC solution was added (AccQ-Tag Ultra Derivatization Kit, Waters), giving a total volume of 50 μl, and mixed thoroughly immediately after addition. After 10 min incubation at 55 °C, samples were loaded onto a Waters UPLC H-Class Plus system with an Acquity UPLC Quaternary Solvent Manager and Sample Manager. Derivatized amino acids were detected on a Waters Acquity QDa single quadrupole mass detector in positive mode. The Qda was operated by one MS, followed by selected ion monitoring (SIR) for each individual amino acid with a defined retention window. The column temperature was maintained at 55 °C. The injection volume was 1 μl. Gradient elution was performed using 0.1% formic acid in water as eluent A and 0.1% formic acid in acetonitrile as eluent B. The flow rate was kept constant at 0.5 m min^–1^ with the following gradient (expressed as solvent B): initial conditions: 1.0% B, 0.0–1 min: 1% B, 1–4 min: 13.0% B, 4–8.5 min: 15.0% B, 8.5–9.5 min: 95.0% B, 9.5–11.5 min: 95% B, 12–15 min: 1% B. The data acquisition and data analysis were done by Masslynx 4.2 (Waters).

### Propidium iodide staining

Propidium iodide (PI) staining was conducted by a 2 min incubation of 5-day-old Arabidopsis seedlings in PI (Sigma Aldrich P4864) diluted to 1 µg ml^–1^ in water. Seedlings were then immediately washed for 3 min and mounted in water. A TCS SP5 laser scanning confocal microscope (Leica) with a ×20 immersion objective (Leica ×20/0.75 NA) was used for imaging. PI was excited at 561 nm with a 30% laser power and detected at 610–620 nm.

After acquisition, images were stitched together to obtain a complete image of the root. Cell lengths were measured manually in Fiji, starting from the beginning of the meristematic region.

### Immunoblotting

*LRX1ΔE14* harbours a cMyc tag at the beginning of the LRR domain, which does not affect protein function and enables protein detection by immunoblotting (Baumberger *et al.*, 2001; Ringli, 2010). For the exact position of the cMyc tag, the sequence of LRX1cMyc is deposited as NCBI accession number GU235992. Root material of 100 seedlings grown for 7 d on half-strength MS plates in a vertical orientation was collected, frozen in liquid nitrogen, and macerated with glass beads. Proteins were extracted using 100 µl of 1% SDS, 5 mM CaCl_2_, and cOmplete Protease Inhibitor Cocktail (Roche). After boiling for 5 min, samples were cooled on ice, centrifuged, and 20 µl of supernatant was mixed with 5 µl of 5× Lämmli buffer containing 5 mM DTT. The samples were boiled at 95 °C for 5 min, then cooled on ice for 3 min, and subsequently centrifuged. A 20 µl aliquot of protein extracts was loaded on a 10% SDS–PAGE gel using standard procedures. Following protein separation, blotting on a polyvinylidene fluoride (PVDF) membrane was done using wet electroblotting (Bio-Rad). After blocking the membrane overnight with 1× Tris-buffered saline (TBS), 0.1% Tween-20, and 5% low-fat milk powder, antibody incubation was done at 1:3000 dilution for both the primary anti-c-Myc (9E10, Thermo Fisher Scientific) and the anti-mouse IgG–horseradish peroxidase (HRP) (Sigma Aldrich A4416) antibodies. Immunodetection was performed using Pierce™ ECL Western Blotting Substrate (Thermo Scientific), and a FUSION FX imager (Vilber) was used for blot visualization.

For the phosphorylation assay, whole seedlings were ground in Eppendorf tubes using a pellet pestle, and total proteins were extracted in SDS extraction buffer [40 mM Tris–HCl pH 7.5, 10% glycerol, 5 mM MgCl_2_, 4% SDS, 1× protease inhibitor cocktail (Sigma-Aldrich), 1 mM phenylmethanesulfonyl fluoride]. Protein concentrations were measured using the Pierce™ BCA Protein assay kit (Thermo Fisher Scientific). A 30 µg aliquot of total protein extracts was mixed with 1/5 volume of 5× Lämmli buffer (250 mM Tris–HCl pH 6.8, 5% SDS, 50% glycerol, 0.02% bromophenol blue), 5% β-mercaptoethanol. Proteins were separated on a 12% SDS––PAGE gel and transferred to PVDF membranes by wet electroblotting (Bio-Rad). To improve signal detection, membranes were treated with the western blot enhancer SuperSignal™ (Pierce). Membranes were then blocked in 5% non-fat dry milk solution in phosphate-buffered saline (PBS; 137 mM NaCl, 2.7 mM KCl, 10 mM Na_2_HPO_4_, 2 mM KH_2_PO_4_, pH7.4) and then probed overnight with either mouse anti-RPS6 (dilution 1:1000, Cell Signaling) or rabbit anti-P-RPS6 (dilution 1:3000) antibodies (Dobrenel *et al.*, 2016b) at 4 °C. Goat anti-rabbit IgG–HRP (Sigma Aldrich A0545) and goat anti-mouse IgG–HRP (Sigma Aldrich A4416) were used as secondary antibodies (dilution 1:5000). Immunodetection was performed using Pierce™ ECL Western Blotting Substrate (Thermo Scientific) or, for lower signals, with SuperSignal™ West Femto Maximum Sensitivity Substrate (Thermo Scientific). A FUSION FX imager (Vilber) was used for blot visualization. Transferred proteins on PVDF membranes were visualized by Coomassie staining to check for equal loading.

### Statistical analyses

Graphs and statistics were generated using R. A Shapiro–Wilk test was used to test the normality of the data. If the normality assumption was not rejected, a Student’s *t*-test was used for mean comparison. If the normality assumption was rejected, the Wilcoxon non-parametric test was used instead. When multiple variances were compared under normality assumption, one-way ANOVA was employed. When seedlings were grown on different plates, a linear model including the plate effect was used. If the data were not normally distributed, a generalized linear model assuming a gamma distribution was employed, as indicated (McCullagh, 2019). In this study, *P*-values <0.05 were considered statistically significant. The significance of the *P*-values remained consistent regardless of the statistical methods used, with the *t*-test and Wilcoxon tests yielding comparable *P*-values for mean comparisons, and the Gamma-GLM and linear models producing similar results for comparisons including a plate effect. The raw data of all the analyses can be found in Supplementary Dataset S1.

## Results

### *sune82* suppresses the *LRX1ΔE14* and *lrx1* root hair phenotypes

**Fig. 1.**
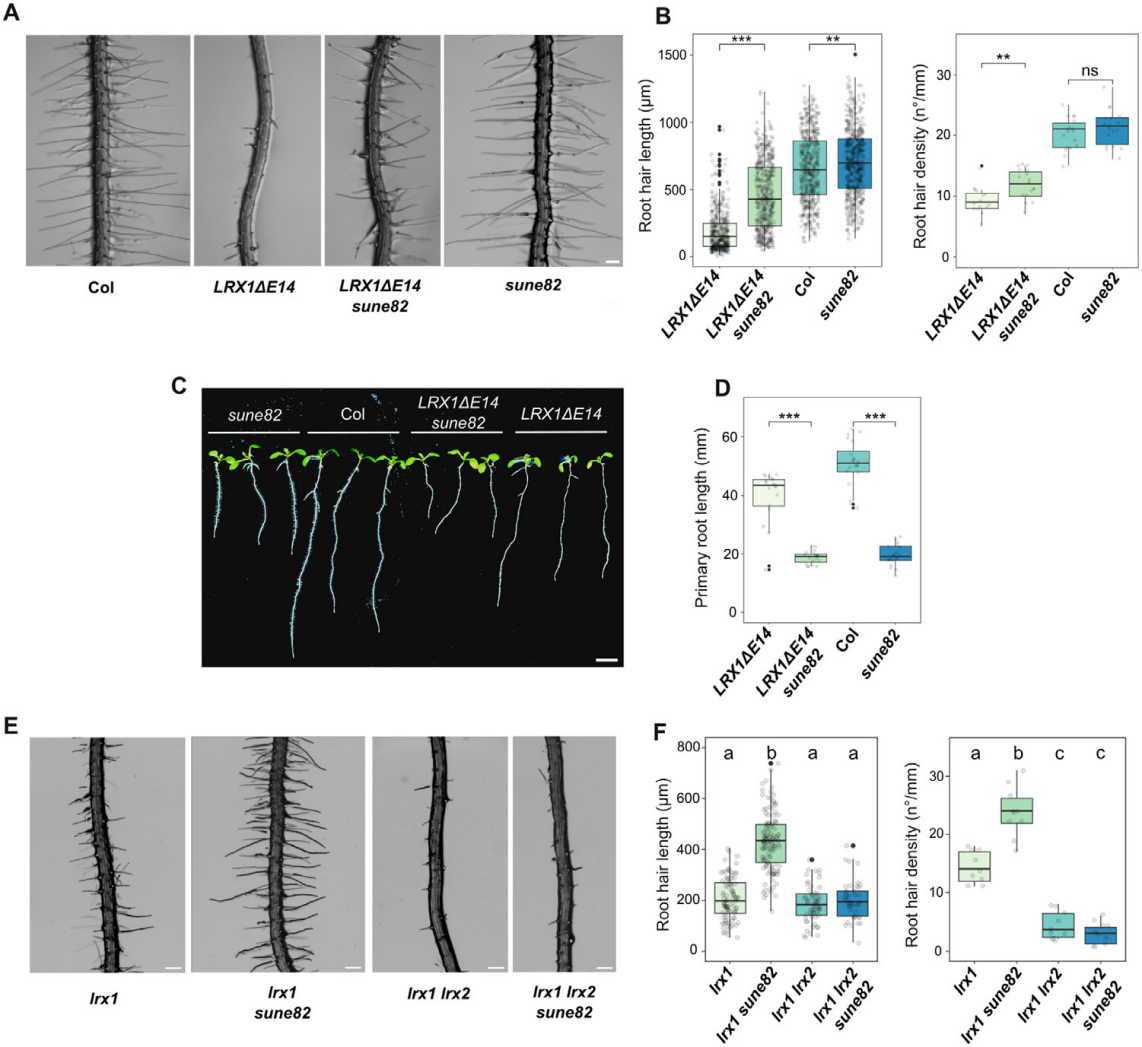
*sune82* suppresses the *LRX1ΔE14* and *lrx1* root hair phenotypes. (A) Five-day-old roots of Col, *LRX1ΔE14*, *LRX1ΔE14 sune82*, and *sune82.* Seedlings were grown in a vertical orientation (scale bar=200 µm). (B) Quantification of root hair length (≥17 roots for each genotype, 30 root hairs were measured per root, *n*≥510) and root hair density (≥17 roots for each genotype, *n*≥17). Statistical analysis was performed using a generalized linear model (Gamma distribution) for root hair length and a linear model for root hair density. (C) Ten-day-old seedlings grown as in (A) (scale bar=5 mm). (D) Primary root length quantification of 10-day-old seedlings (≥20 roots for each genotype, *n*≥20). Statistical analysis was performed using a linear model. Asterisks on the graphs indicate significant differences between genotypes (*P<0.05, **P<0.01, ***P<0.001), and ns indicates non-significant differences (*P*>0.05). The black line in the boxplots represents the median. (E) Five-day-old roots of *lrx1*, *lrx1 sune82*, *lrx1 lrx2*, and *lrx1 lrx2 sune82.* The *lrx1 lrx2* mutant develops a stronger root hair defect than *lrx1* (scale bar=200 µm). (F) Quantification of root hair length (≥9 roots for each genotype, ≥10 root hairs were measured per root, *n*≥100) and root hair density (≥9 roots for each genotype, *n*≥9). The letters indicate statistical significance (one-way ANOVA followed by post-hoc Tukey’s HSD, α=0.05).

As expression in wild-type Col of a truncated LRX1 lacking its extensin domain (*LRX1ΔE14*) leads to a dominant-negative effect (Baumberger *et al.*, 2001; Ringli, 2010) (Fig. 1A, B), we took advantage of this striking root hair phenotype in a genetic attempt to identify new components able to modify the LRX1-modified process. A transgenic line expressing *LRX1ΔE14* (Ringli, 2010) was used for EMS mutagenesis. Progenies of 1000 M_1_ plants were screened for suppression of the *LRX1ΔE14*-induced root hair growth defect (for details, see the Materials and methods). Twenty-two suppressors called *suppressor of dominant-negative effect* (*sune*) mutants were identified, and one line, *sune82*, was analyzed in detail. This line, referred to as *LRX1ΔE14 sune82*, exhibits a partial suppression of the *LRX1ΔE14* root hair phenotype (Fig. 1A) with more and longer root hairs developing (Fig. 1B). *LRX1ΔE14 sune82* also displays a severe dwarf phenotype with shorter primary roots (Fig. 1C, D) and stunted adult plants (Supplementary Fig. S1) compared with *LRX1ΔE14*. Backcrossing of *LRX1ΔE14 sune82* with the parental *LRX1ΔE14* line produced F_1_ seedlings with an *LRX1ΔE14* phenotype, indicating that the *sune82* mutation is recessive. A segregation analysis of the *sune82* mutant phenotype revealed 3:1 segregation of the wild type:*sune82* phenotype (609 wild type:196 *sune82*), confirming that *sune82* is a recessive mutation.

To determine the effect of the *sune82* mutation in the wild-type background, the *LRX1ΔE14 sune82* mutant was crossed with wild-type Col. In the F_2_ generation, plants with a short-root *sune82* mutant phenotype but lacking *LRX1ΔE14* were selected. *sune82* single mutants develop significantly longer root hairs than Col (Fig. 1A, B) and a short primary root comparable with *LRX1ΔE14 sune82* (Fig. 1C, D).

We then wanted to investigate whether *sune82* is capable of suppressing *lrx1*, which exhibits an intermediate root hair defect. To this end, *sune82* was crossed with the *lrx1* mutant, and an *lrx1 sune82* double mutant was selected in the F_2_ generation. Double mutants showed suppression of the *lrx1* root hair defect, with a higher frequency of root hair formation and longer root hairs compared with *lrx1* (Fig. 1E, F). We also tested whether the *sune82* mutation is able to suppress the *lrx1 lrx2* double mutant, in which both Arabidopsis root hair-expressed *LRX* genes are mutated, resulting in a severe root hair phenotype (Baumberger *et al.*, 2003) analogous to that of *LRX1ΔE14*. The *lrx1 lrx2 sune82* triple mutant developed the typical *sune82* short-root phenotype; however, the *lrx1 lrx2* double mutant phenotype was not suppressed by *sune82* (Fig. 1E, F). These results suggest that the *sune82*-mediated enhancement of root hair growth is dependent on LRX proteins: while a partial disruption of the LRX pathway results in root hair defects that can be compensated by the *sune82* mutation, a complete disruption of the LRX pathway cannot be compensated for. Additionally, the stunted adult plant phenotype and the reduction in primary root length demonstrate that the effect of the *sune82* mutation is widely involved in cell growth and does not solely influence the LRX1-related root hair developmental process.

### The *sune82* mutation affects the *HISN2* gene involved in His biosynthesis

**Fig. 2.**
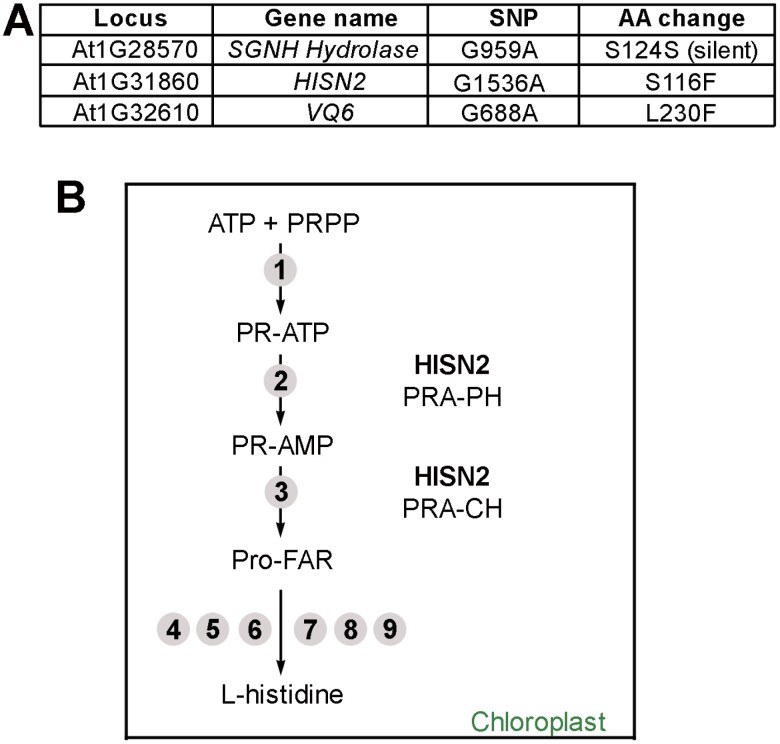
The *sune82* mutation affects the *HISN2* gene involved in His biosynthesis. (A) Three linked SNPs in coding sequences were identified in *LRX1ΔE14 sune82* compared with the non-mutagenized *LRX1ΔE14* line, of which the SNP in *HISN2* showed complete linkage. (B) HISN2 catalyzes the second and third steps of histidine biosynthesis in the chloroplast. Numbers indicate individual enzymatic steps in the pathway; those conducted by HISN2 are mentioned: No. 2, PRA-PH, phosphoribosyl-ATP pyrophosphatase, and No. 3, PRA-CH, phosphoribosyl-AMP cyclohydrolase.

To identify the *sune82* mutation, F_2_ plants of the backcross *LRX1ΔE14 sune82*×*LRX1ΔE14* that showed a *sune82*-like phenotype were selected and pooled, and DNA was extracted for whole-genome sequencing, along with DNA of the non-mutagenized *LRX1ΔE14*. Homozygous mutations specific to the suppressor lines and absent from the parental *LRX1ΔE14* line were retained, and nucleotide changes in coding sequences were considered. Three genetically linked mutations in coding sequences on chromosome 1 were identified (Fig. 2A). A co-segregation analysis was conducted on individual seedlings of the segregating F_2_ population using CAPS markers developed for the three identified SNPs (Supplementary Fig. S2A). This analysis revealed that the mutation in *HISN2*, but not the other SNPs, is completely linked to the *sune82* phenotype.

*HISN2* (*AT1G31860*) encodes a bifunctional enzyme involved in His biosynthesis and is expressed in various tissues (Fig. 2B; Supplementary Fig. S2B). HISN2 catalyzes the second and third steps in His biosynthesis in the chloroplast via two distinct domains: phosphoribosyl-ATP pyrophosphatase (PRA-PH) and phosphoribosyl-AMP cyclohydrolase (PRA-CH) (Fig. 2B). PRA-PH catalyzes the second step in which *N*1-5ʹ-phosphoribosyl-ATP (PR-ATP) is hydrolyzed to *N*1-5ʹ-phosphoribosyl-AMP (PR-AMP), while PRA-CH opens the adenine ring of PR-AMP to produce *N*1‐[(5ʹ‐phosphoribosyl)formimino]‐5‐aminoimidazole‐4‐carboxamide‐ribonucleotide (ProFAR). Six additional enzymes then convert this intermediate to His (Witek *et al.*, 2021).

### Histidine biosynthesis is reduced in *sune82* plants

**Fig. 3.**
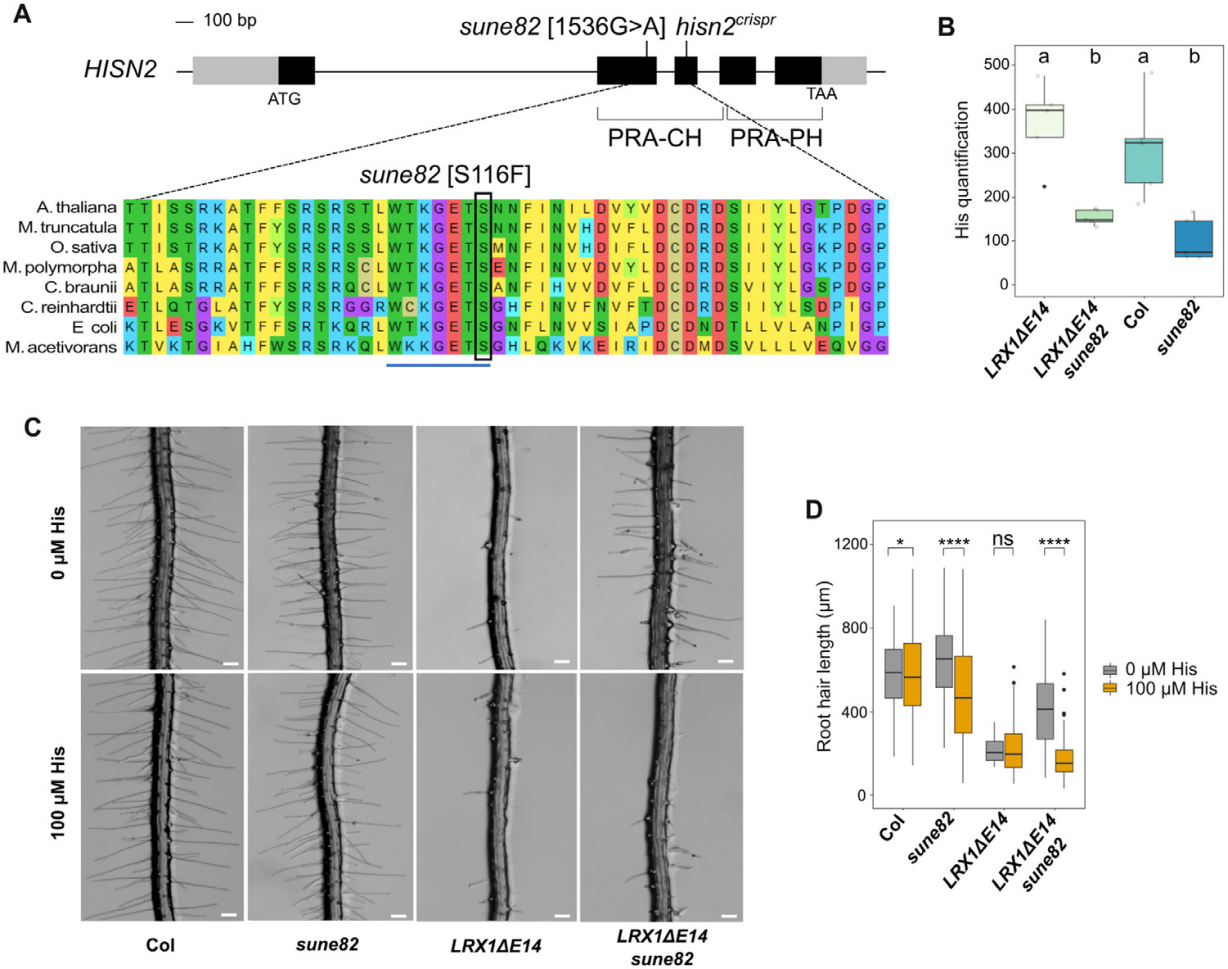
The *sune82* mutation changes the active site of *HISN2*, leading to a reduced histidine production. (A) Genomic structure of *HISN2* (AT1G31860) with the *sune82* mutation at position 1536 (guanine to adenine substitution) relative to the start codon indicated and amino acid alignment with its orthologs using MUSCLE in MEGA11. Gray boxes=untranslated regions, black boxes=exons, lines=introns. PRA-CH, phosphoribosyl-AMP cyclohydrolase; PRA-PH, phosphoribosyl-ATP pyrophosphatase. *Arabidopsis thaliana*, *Medicago truncatula*, *Oryza sativa*, *Marchantia polymorpha*, *Chara braunii, Chlamydomonas reinhardtii*, *Escherichia coli*, and *Methanosarcina acetivorans*. The Ser116 residue altered by the *sune82* mutation is boxed. (B) His levels were determined by LC-MS in 10-day-old seedlings. Arbitrary units show that His content is reduced by the *sune82* mutation. The letters indicate statistical significance (one-way ANOVA followed by post-hoc Tukey’s HSD, α=0.05). The black line in the boxplots represents the median. (C) Five-day-old roots of Col, *LRX1ΔE14*, *LRX1ΔE14 sune82*, and *sune82* grown with or without 100 µM His. Scale bars=200 μm. (D) Quantification of root hair length [≥5 roots for each genotype, ≥15 root hairs were measured per root (when present), *n*≥60]. Statistical analysis was performed using a generalized linear model (Gamma distribution). Asterisks on the graphs indicate significant differences (*P<0.05, ****P<0.0001), and ns indicates non-significant differences (*P*>0.05).

*LRX1ΔE14 sune82* plants contain a G-to-A substitution in *HISN2* causing [S116F] substitution in the PRA-CH domain (Fig. 3A; Supplementary Fig. 3A). The crystal structure of HISN2 has recently been determined in *Medicago truncatula* (Witek *et al.*, 2021). In the MtHISN2-AMP complex, PR-AMP interacts with the PRA-CH domain in a specific region formed by residues _107_WTKGETS_113_. Amino acid alignment of diverse species shows that this PR-AMP-binding region is highly conserved throughout all domains of life (Fig. 3A). Remarkably, Witek *et al.* (2021) observed that substitution of the polar amino acid Ser in this motif with Ala (MtS113A) reduces the activity of MtHISN2. Hence, the substitution of the corresponding position in the Arabidopsis HISN2 (AtS116F) in *sune82* could potentially reduce the activity of HISN2.

To determine the impact of the *sune82* mutation on His production, His levels were determined by LC-MS in 10-day-old seedlings. *sune82* and *LRX1ΔE14 sune82* lines exhibited a 58% and 67% reduction in His content compared with wild-type Col and *LRX1ΔE14*, respectively (Fig. 3B). It is noteworthy that a comparable reduction was observed for the activity of HISN2_S113A_ versus HISN2_WT_ in *M. truncatula* (Witek *et al.*, 2021), suggesting that the AtS116F substitution affects HISN2 activity to a similar degree.

We next wanted to confirm that the lack of His was responsible for the suppression of the *LRX1ΔE14* phenotype. *LRX1ΔE14* and *LRX1ΔE14 sune82* seedlings were germinated on media supplemented with or without 100 µM His. When grown on 100 µM His, the root hair phenotype of *LRX1ΔE14 sune82* seedlings was comparable with *LRX1ΔE14* (Fig. 3C, D), with shorter and burst root hairs, indicating that His supplementation can compensate for the *sune82* mutation. The short-root phenotype of *sune82* plants could also be suppressed by His supplementation (Supplementary Fig. S3B, C), confirming that the reduced His content causes the dwarf phenotype and the suppression of *LRX1ΔE14*.

### A *HISN2* knockout mutant is lethal

**Fig. 4.**
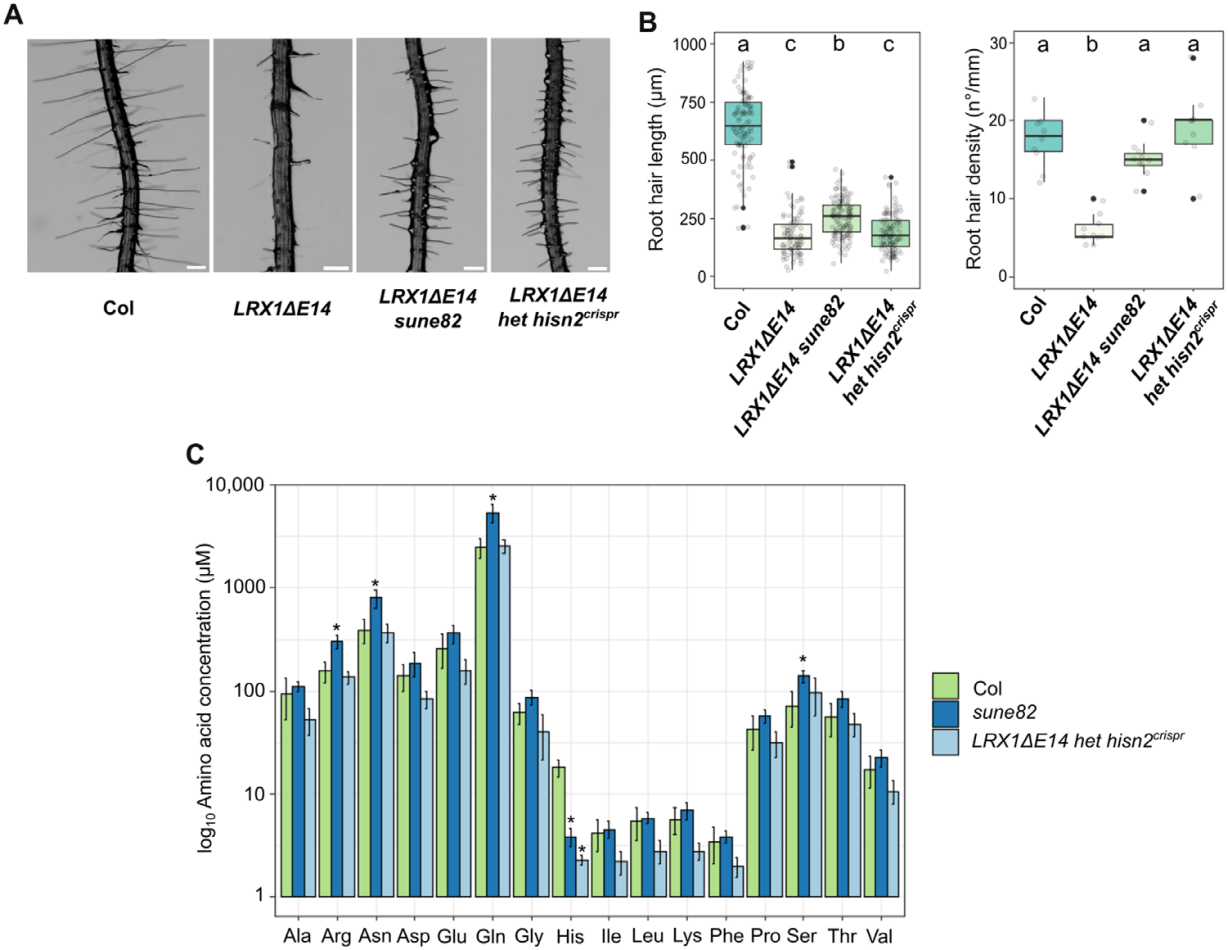
Heterozygous *hisn2*^*crispr*^ seedlings suppress the *LRX1ΔE14* root hair phenotype. (A) Four-day-old roots of seedlings of wild-type Col, *LRX1ΔE14*, *LRX1ΔE14 sune82*, and *LRX1ΔE14* heterozygous for *hisn2*^*crispr*^. Scale bars=200 μm. (B) Quantification of root hair length (≥ 9 roots for each genotype, ≥10 root hairs were measured per root, *n*≥100) and root hair density (≥9 roots for each genotype, *n*≥9). The letters indicate statistical significance (one-way ANOVA followed by post-hoc Tukey’s HSD, α=0.05). (C) Analysis of the free amino acid content of wild-type Col, *sune82*, and heterozygous *hisn2*^*crispr*^. Note the log_10_ scale on the *y*-axis. Absolute values are displayed in Supplementary Dataset S2. Error bars indicate the SD. Asterisks on the graph indicate significant differences from Col (*P<0.05, *t*-test).

To further confirm that mutations in *HISN2* confer the suppressor phenotype in *LRX1ΔE14 sune82*, we generated a knockout allele of *HISN2* in the *LRX1ΔE14* background using CRISPR/Cas9 technology. A guide RNA targeting the third exon of *HISN2*, located in the PRA-CH coding domain, was used to obtain a *hisn2*^*crispr*^ allele containing an A insertion, leading to a preliminary stop codon at the end of the PRA-CH domain (Fig 3A; Supplementary Fig. S4A). It is known that His is essential for plant survival and that a complete blocking of His biosynthesis is embryo lethal (Muralla *et al.*, 2007). When the progeny of a heterozygous *hisn2*^*crispr*^ mutant was sown, seedlings with shorter roots were identified, and sequencing revealed these to be heterozygous for the *hisn2*^*crispr*^ mutation (Supplementary Fig. S4A). Importantly, heterozygous *hisn2*^*crispr*^ seedlings showed suppression of the *LRX1ΔE14* root hair phenotype, producing root hairs that were significantly shorter than in *LRX1ΔE14 sune82* (Fig. 4A, B). Heterozygous *hisn2*^*crispr*^ mutants exhibited a variable degree of growth defects (Supplementary Fig. S4B), some requiring His supplementation for survival (Supplementary Fig. S4C), suggesting haplo-insufficiency (Deutschbauer *et al.*, 2005). Additionally, heterozygous *hisn2*^*crispr*^ plants exhibited a dwarf phenotype at the adult stage comparable in size with *sune82* (Supplementary Fig. S4D). No homozygous *hisn2*^*crispr*^ line could be identified among these seedlings. These data show that *hisn2*^*crispr*^ is a recessive, lethal mutation and that *hisn2*^*crispr*^ heterozygous plants probably have reduced HISN2 activity. To investigate this point further, seeds of the segregating population that did not germinate on regular MS were supplemented with 100 µM His. Under these conditions, germination was possible, and a few of these seedlings were revealed to be homozygous for the *hisn2*^*crispr*^ mutation. This confirms the importance of His synthesis for plant growth and development, and demonstrates the lethality of a *hisn2* knockout allele (Muralla *et al.*, 2007).

To test the overall impact on amino acid accumulation of the mutants in *HISN2*, a free amino acid quantification was conducted in wild-type, *sune82*, and the *hisn2*^*crispr*^ lines. The *HISN2*^*crispr*^ allele is in the *LRX1ΔE14* genetic background; however, considering the restriction of *LRX1ΔE14* expression in root hairs representing a small fraction of cells in a seedling, it can be assumed that any change in *LRX1ΔE14 hisn2*^*crispr*^ can be attributed to *hisn2*^*crispr*^. The histidine content of *sune82* and *LRX1ΔE14 hisn2*^*crispr*^ was found to be significantly reduced in comparison with wild-type Col, with a 79% reduction in His content for *sune82* and 87% for *hisn2*^*crispr*^ (Fig. 4C; Supplementary Dataset S2). Interestingly, levels of Arg (+48%), Asn (+50%), Gln (+54%), and Ser (+49%) were found to be significantly elevated in the *sune82* mutant when compared with Col, but not in the heterozygous *hisn2*^*crispr*^ mutant.

### Reduced HISN2 activity leads to an altered TOR sensitivity

**Fig. 5.**
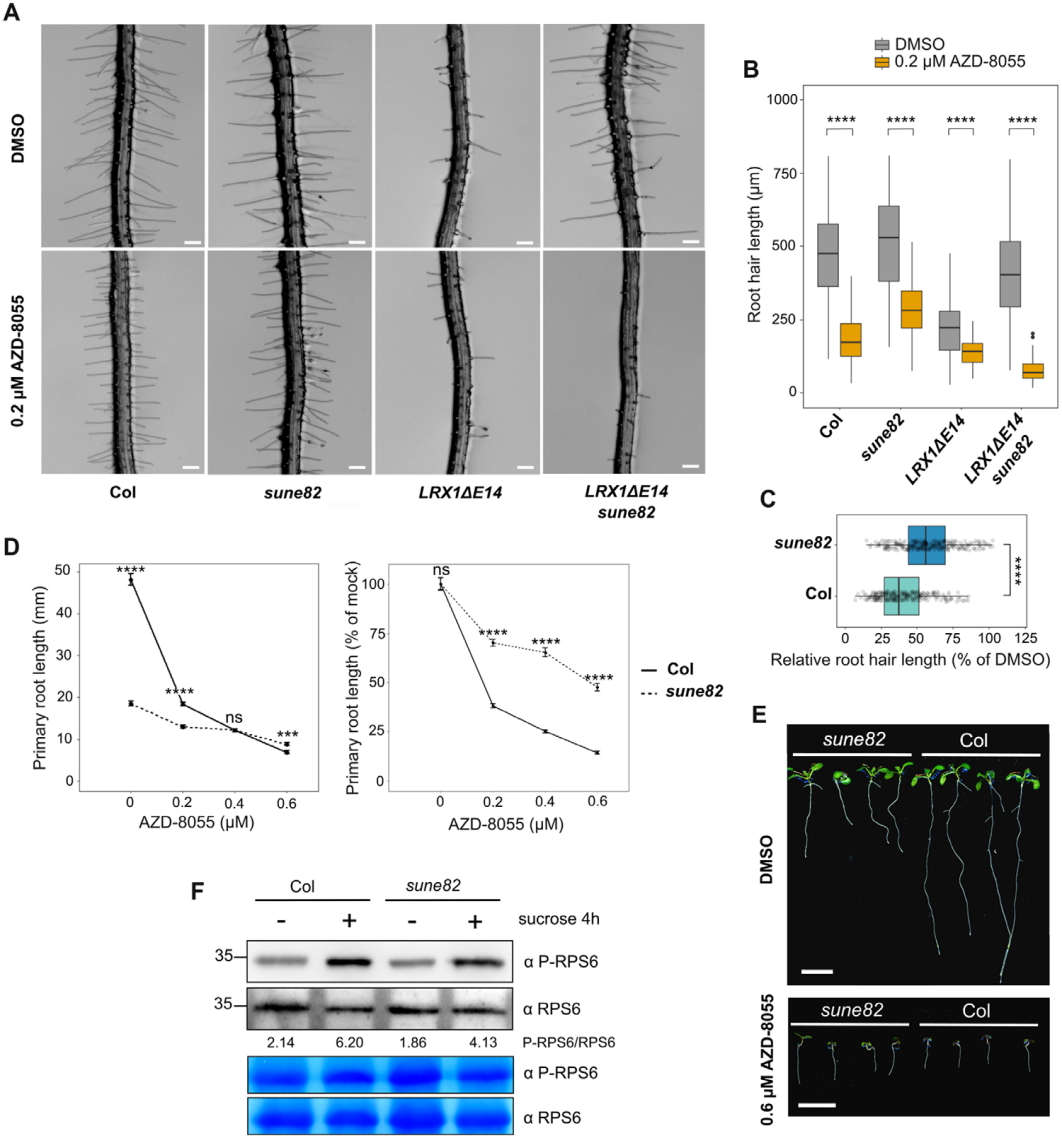
Reduced HISN2 activity leads to an altered TOR sensitivity. (A) Five-day-old roots of Col, *sune82, LRX1ΔE14*, and *LRX1ΔE14 sune82* grown with DMSO (mock) or 0.2 µM AZD-8055. Scale bars=200 µm. (B) Quantification of root hair length [≥9 roots for each genotype, ≥10 root hairs were measured per root (when present), *n*≥40]. Statistical analysis was performed using a generalized linear model (Gamma distribution). Asterisks on the graphs indicate significant differences (****P<0.0001). (C) Reduction in root hair length (0.2 µM AZD-8055/DMSO) as a percentage. AZD-8055 caused stronger reduction in root hair length in Col than in *sune82*. Data shown in (B) were used. Asterisks on the graphs indicate significant differences (****P<0.0001, Wilcoxon test). (D) Primary root length quantification of 9-day-old seedlings grown at different concentrations of AZD-8055. The first panel displays the absolute primary root length (mm), and the second panel shows relative root length compared with mock conditions. Asterisks on the graph indicate significant differences between genotypes (*n*≥20, ****P<0.0001, ***P<0.001, ns *P*>0.05 and not significant, linear model). (E) Nine-day-old seedlings grown on 0.6 µM AZD-8055. The primary root length of Col is significantly reduced, while that of *sune82* is almost unaffected. Scale bars=1 cm. (F) For RPS6 analysis, 6-day-old seedlings were transferred to sugar-free medium for 24 h and then either mock or sucrose (0.5%) treated for 4 h to induce RPS6 phosphorylation. A 30 µg aliquot of whole-seedling extracts was subjected to immunoblot analysis with anti-phospho-RPS6 Ser240 or anti-total RPS6 antibodies, and the anti-phospho-RPS6 Ser240/anti-total RSP6 ratios are indicated. Coomassie blue to ensure equal protein loading is also shown.

Amino acid levels have been demonstrated to directly influence the TOR network, which coordinates plant growth (Cao *et al.*, 2019; Liu *et al.*, 2021; Mallén-Ponce *et al.*, 2022). In particular, His, Gln, Ser, and Asn have been shown to activate TOR when exogenously supplied to inorganic nitrogen-starved seedlings (Liu *et al.*, 2021). Therefore, we hypothesized that the alterations in *sune82* amino acid metabolism would affect TOR activity, leading to a change in plant growth. To first test whether TOR activity is impacted, we employed a specific TOR kinase inhibitor, AZD-8055, that targets the TOR kinase. Sensitivity to TOR inhibitors is frequently used to identify modulations in the TOR network that influence TOR kinase activity (Chan *et al.*, 2000; Leiber *et al.*, 2010; Barrada *et al.*, 2019; Schaufelberger *et al.*, 2019). Treatment of wild-type seedlings with AZD-8055 inhibits cell growth and root hair elongation (Montané and Menand, 2013). In the presence of AZD-8055, suppression of the *LRX1ΔE14* phenotype mediated by the *sune82* mutation was abolished (Fig. 5A, B). This indicates that TOR kinase activity is involved in the suppression of the *LRX1ΔE14* root hair phenotype. To determine whether the *sune82* mutant has an altered sensitivity to AZD-8055 treatment, *sune82* and wild-type seedlings were germinated at different concentrations of AZD-8055. Already when applied at a low concentration (0.2 µM), AZD-8055 treatment decreased root hair length in Col and *sune82* (Fig. 5A, B). However, the impact of AZD-8055 treatment on root hair development differed significantly between Col and *sune82* (Fig. 5C). While AZD-8055 treatment resulted in a 60% reduction in root hair length in Col, this reduction was only 42.5% in *sune82*. This suggests that *sune82* is more resistant to AZD-8055 treatment in root hairs. Next, the impact of AZD-8055 on primary root growth was assessed. *sune82* seedlings were strikingly less affected by AZD-8055 treatment than the wild type at all concentrations (Fig. 5D). Already at a low concentration (0.2 μM) of AZD-8055, *sune82* seedlings presented a decrease of only ~30% in primary root length compared with mock conditions, whereas wild-type primary root length showed a decrease of 60%. At high concentration (0.6 µM) of AZD-8055, *sune82* exhibited a decrease of ~50% in primary root length, while wild-type primary root length demonstrated a decrease of 90% (Fig. 5D, E). *LRX1ΔE14 sune82* seedlings also presented a reduced AZD-8055 sensitivity compared with *LRX1ΔE14* regarding root length (Supplementary Fig. S5A, B). Strikingly, growing seedlings for a longer period on 0.6 µM AZD-8055 revealed that *sune82* and *LRX1ΔE14 sune82* seedlings developed longer primary roots than the wild type and *LRX1ΔE14*, respectively (Supplementary Fig. S5C). These results indicate that the TOR network is affected in the *sune82* mutant.

To test whether the reduction in histidine content in *sune82* is solely responsible for the observed resistance to the TOR inhibitor AZD-8055, seedlings were grown on plates containing 0.6 µM AZD-8055, supplemented or not with 100 µM His. The addition of His resulted in a notable increase in primary root length in both Col and *sune82*, with a particularly pronounced effect observed in *sune82* (Supplementary Fig. S5D, E), yet the AZD-8055 sensitivity remained the same. These findings suggest that the resistance to the TOR inhibitor AZD-8055 in *sune82* seedlings is not exclusively attributable to the change in His levels, but rather to the sum of all changes in amino acid metabolism.

The TOR complex promotes cell proliferation, and its inactivation leads to a reduction in meristem size and induces early differentiation (Ren *et al.*, 2012; Cao *et al.*, 2019). To determine whether *sune82* has an altered meristem size, root meristems of *sune82* and Col seedlings were examined using PI staining for visualization of the individual cells. *sune82* seedlings showed a reduction in meristem size and cell number, and the transition zone (TSZ), which separates dividing cells from elongating cells into two functional domains, appeared closer to the root tip than in Col (Supplementary Fig. S5F, G). Consequently, the number of cells required to reach a root length of 500 µm was lower in *sune82* than in Col (Supplementary Fig. S5H). This suggests that reduced cell proliferation in the meristematic region contributes to the reduced primary root length observed in seedlings containing the *sune82* mutation.

Given the reduced sensitivity to the TOR inhibitor AZD-8055 and the short meristem size and primary root length of the *sune82* mutant, we investigated whether TOR activity was decreased at the seedling stage, using phosphorylation of ribosomal protein S6 (RPS6^S240^) as a readout. RPS6 is a downstream effector of TOR that is commonly used to measure TOR activation in Arabidopsis (Ren *et al.*, 2012; Dobrenel *et al.*, 2016b; Forzani *et al.*, 2019; Mallén-Ponce *et al.*, 2022). Antibodies against RPS6 and phosphorylated RPS6, respectively, that allow assessment of phosphorylation levels, revealed no obvious difference between the wild type and *sune82* mutant (Fig. 5F). In addition, when TOR activity was induced by sucrose treatment, RPS6 phosphorylation increased to similar levels in *sune82* and wild-type seedlings. This result indicates that the RPS6 pathway is not altered in *sune82*.

Taken together, these data indicate that the *sune82* phenotype is associated with a modified TOR network, but RPS6 phosphorylation seems not to be a major target of this altered activity.

### Alleles of *IPMS1* suppress *LRX1ΔE14*

**Fig. 6.**
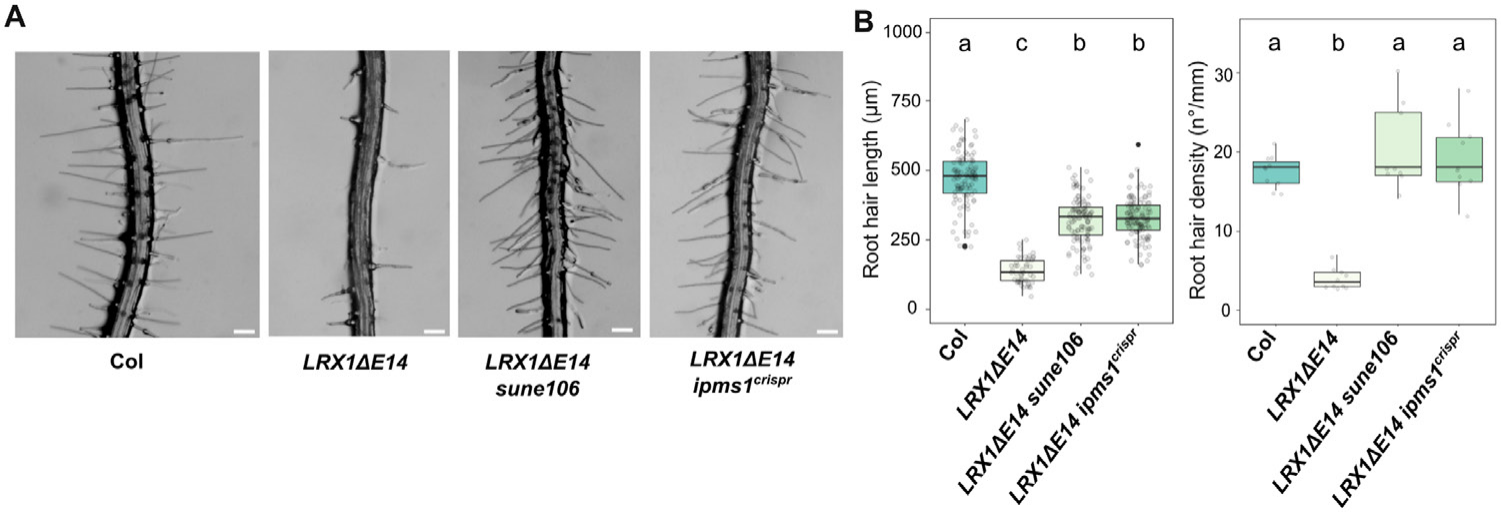
*sune106* suppresses the *LRX1ΔE14* root hair phenotype. (A) Five-day-old roots of Col, *LRX1ΔE14*, *LRX1ΔE14 sune106*, and *LRX1ΔE14 ipms1*^*crispr*^ show suppression of the *LRX1ΔE14* phenotype by mutations in *IPMS1*. Scale bars=200 µm. (B) Quantification of root hair length [≥9 roots for each genotype, ≥10 root hairs were measured per root (when present), *n*≥40] and root hair density (≥9 roots for each genotype, *n*≥9). The letters indicate statistical significance (one-way ANOVA followed by post-hoc Tukey’s HSD, α=0.05).

The finding of a suppressor of *LRX1ΔE14* associated with the TOR pathway prompted us to investigate whether other known modifiers would also be found in the *sune* mutant collection. Mutations in *IPMS1*, an enzyme required for Leu biosynthesis, affect amino acid homeostasis and alter the TOR network (Cao *et al.*, 2019; Schaufelberger *et al.*, 2019). Consequently, the *IPMS1* locus of all *sune* mutants initially identified and subsequently confirmed was sequenced. One line, *sune106,* was found to have a G-to-A mutation in *IPMS1*, resulting in a [G99D] substitution in a highly conserved stretch of amino acids (Supplementary Fig. S6A, B). To confirm the causative effect of the mutation in *IPMS1*, an additional mutation was introduced in *IPMS1* in the *LRX1ΔE14* background using CRISPR/Cas9 technology. A guide RNA targeting a sequence adjacent to the SNP of the *sune106* allele was utilized, and an *LRX1ΔE14* line homozygous for an insertion of an adenine leading to a premature stop codon was produced (Supplementary Fig. S6A). This *ipms1*^*crispr*^ line also showed suppression of the *LRX1ΔE14* root hair phenotype (Fig. 6), corroborating that the causative mutation in *sune106* is in *IPMS1*. Hence, modulating the TOR network by interfering with the Leu biosynthetic pathway also causes suppression of the *LRX1ΔE14* phenotype.

## Discussion

### The *sune82* mutation leads to a partial activity of HISN2

His is an essential amino acid that is required for plant growth and reproduction (Mo *et al.*, 2006; Muralla *et al.*, 2007). In contrast to the majority of amino acids which are produced by enzymes encoded by multi-gene families, five of the eight His biosynthesis enzymes are encoded by single-copy genes in Arabidopsis (Muralla *et al.*, 2007; Ingle, 2011). Knockout mutations in most of these genes therefore have lethal effects (DeFraia and Leustek, 2004; Tzafrir *et al.*, 2004; Muralla *et al.*, 2007; Boavida *et al.*, 2009; Petersen *et al.*, 2010; Meinke, 2020). To date, two viable weak alleles of His biosynthesis genes have been identified in Arabidopsis. The *apg10* mutant carries a [V256L] mutation in *HISN3* and exhibits a pale green phenotype in seedlings. Unlike embryo-lethal *hisn3* knockout mutants, *apg10* plants gradually recover and are wild type-like in reproductive tissues (Noutoshi *et al.*, 2005). The *hpa1* mutant presents a [A69T] substitution in *HISN6A*, which results in impaired root development in seedlings. Nevertheless, adult plants are indistinguishable from the wild type, potentially due to a gain in expression of its paralog *HISN6B* (Mo *et al.*, 2006). Interestingly, *apg10* and *hpa1* have different effects on His content. The *apg10* mutation does not reduce free His content compared with the wild type, but leads to a general increase in amino acid biosynthesis (Noutoshi *et al.*, 2005). *hpa1* mutant seedlings exhibit a 30% reduction in free His content and also show lower levels of free Asp, Lys, Arg, and Glu (Mo *et al.*, 2006). Here, we characterized a novel His-deficient mutant, which presents a 60–80% decrease in free His content and elevated levels of free Arg, Asn, Gln, and Ser. The *sune82* mutant exhibits altered development at both the seedling and adult stage, and an overall dwarf phenotype (Fig. 1; Supplementary Fig. S1). Therefore, *sune82* represents the first viable *HISN* mutant identified with a reduced His content and a mutant phenotype in many tissues and at different developmental stages.

The missense mutation [S116F] in *sune82* affects a residue localized in a highly conserved motif that is directly involved in AMP binding. This has been demonstrated in a detailed structural analysis of the HISN2 enzyme of *M. truncatula* (MtHISN2), where Ser113 corresponds to Ser116 of HISN2 of Arabidopsis (Fig. 3A). Interestingly, Ser113 of MtHISN2 is the sole residue in this motif that can be modified without completely losing enzymatic activity (Witek *et al.*, 2021). The *sune82* mutant thus provides *in vivo* evidence that changing the polar Ser116 to a hydrophobic amino acid alters HISN2 activity, confirming the *in vitro* findings of Witek *et al.* (2021). Therefore, Ser116 in HISN2 may be one of a few residues of HISN2 that can result in an intermediate enzymatic activity and, consequently, a significant but non-lethal growth phenotype. In addition, the amino acids Arg, Asn, Gln, and Ser are increased in abundance in *sune82* (Fig. 4), which in part can be explained by redirecting the metabolic flux away from the inefficient HISN2 towards these other amino acids, some of which share parts of the biosynthetic pathway with His (Yang *et al.*, 2020). The *hisn2*^*crispr*^ allele only shows reduced His levels (Fig. 4), indicating that the reduced His content is the main cause of suppression of *LRX1ΔE14* in *hisn2*^*crispr*^. Apparently, the reduced amount of wild-type HISN2 protein in the heterozygous *hisn2*^*crispr*^ allele has a different effect on metabolism compared with the impaired HISN2 protein encoded by *sune82*. As *LRX1ΔE14 sune82* produces longer root hairs than *LRX1ΔE14 hisn2*^*crispr*^, this difference might be based on the observed discrepancy in the amino acid profile observed for the two *hisn2* alleles (Fig. 4).

### *sune8*2 can suppress the *LRX1ΔE14*-induced root hair growth defect in a TOR-dependent manner

The *sune82* mutation results in enhanced root hair development and suppression of the dominant-negative effect induced by *LRX1ΔE14*, which is dependent on an active TOR network (Fig. 5). As in yeast and in animal cells, the plant TOR network senses nutrient availability to adjust cell growth (Robaglia *et al.*, 2012; Liu *et al.*, 2021; Li *et al.*, 2023). TOR influences the accumulation of sugars such as raffinose, amino acids, and a number of secondary metabolites (Moreau *et al.*, 2012; Ren *et al.*, 2012), and in this way regulates cell wall remodeling and cell growth (Calderan-Rodrigues and Caldana, 2024). *sune82* seedlings are more resistant to the TOR kinase inhibitor AZD-8055 than the wild type, and root hair growth is more pronounced, in contrast to the reduced root growth. Divergent cell type-specific responses to the same prevailing conditions are found for root and root hair growth under nutrient-limited conditions (Vissenberg *et al.*, 2020), where root hair growth is stimulated in a TOR-dependent manner (Pacheco *et al.*, 2023). Several amino acids serve as activators of TOR, including His, Gln, and Ser (Liu *et al.*, 2021). At the same time, inhibition of the TOR kinase leads to changes in metabolic flux and accumulation of Gln and Asn (Ingargiola *et al.*, 2023). This complicates the understanding of the influence of *sune82* on the TOR network. Nevertheless, the similarity of *sune82* to mutations in *IPMS1* (Fig. 6) that impact the TOR network (Cao *et al.*, 2019; Schaufelberger *et al.*, 2019) and to the effects of TOR inhibition on early meristematic cells differentiation (Supplementary Fig. S5) (Montané and Menand, 2013) supports the view that *sune82* has an effect on the TOR network.

Our analysis on the phosphorylation dynamics of RPS6, a ribosomal protein phosphorylated by the S6 kinase 1 (S6K1) that is a direct target of the TOR kinase (Mahfouz *et al.*, 2006), did not reveal significant changes in phosphorylation in *sune82* compared with the wild type. It is plausible that the alterations by *sune82* are rather subtle, while changes in RPS6 phosphorylation are mainly seen when the TOR network is strongly influenced, as exemplified by the treatment with the TOR kinase inhibitor AZD-8055 or by silencing *TOR* expression (Dobrenel *et al.*, 2016b). Compared with these severe treatments that eventually are lethal, the *sune82* is viable and thus appears to have a moderate impact on TOR. The TOR kinase is involved in numerous processes (Ingargiola *et al.*, 2020), and it is possible that downstream effectors are subtly altered, the sum of which results in the observed growth alteration in *sune82*, which are all reminiscent of but weaker than inhibiting the TOR network.

The CrRLK1L receptor kinase FER was recently shown to directly phosphorylate and activate the TOR kinase in a RALF-enhanced manner (Pearce *et al.*, 2001; Song *et al.*, 2022; Pacheco *et al.*, 2023; Liu *et al.*, 2024). This finding establishes a link between the TOR network and the FER-dependent cell growth control machinery that involves LRX proteins. LRXs bind RALF peptides (Mecchia *et al.*, 2017; Moussu *et al.*, 2020), influence the compaction of pectin, a major component of the cell wall (Moussu *et al.*, 2023; Schoenaers *et al.*, 2024), and function in conjunction with FER to regulate a number of processes, including development and cell growth (Dünser *et al.*, 2019; Herger *et al.*, 2019; Gronnier *et al.*, 2022). Since *LRX1ΔE14* lacks the extensin domain that anchors LRX proteins in the cell wall (Rubinstein *et al.*, 1995; Baumberger *et al.*, 2001; Ringli, 2010), it might form inadequate LRX–RALF–pectin connections and disturb FER-mediated signaling that also influences the TOR network. This provides an explanation as to why LRX1-related phenotypes are influenced by a modification of the TOR network (Baumberger *et al.*, 2001; Leiber *et al.*, 2010; Schaufelberger *et al.*, 2019). In the *lrx1* mutant, LRX2 is still present, providing a biologically sufficiently functional LRX–FER axis, causing suppression of *lrx1* by *sune82*. The absence of LRX1 and LRX2, however, abolishes the LRX–FER connection, preventing suppression of the *lrx1 lrx2* double mutant by *sune82* (Fig. 1E).

In conclusion, we have identified a weak allele in a gene involved in His biosynthesis, that results in a mutant with consistent alterations in plant development. In contrast to other mutants affected in this pathway, the *sune82* mutant phenotype is neither lethal nor restricted to a short period during the plant life cycle. This weak *HISN2 sune82* allele allowed establishment of a link between an *LRX1ΔE14*-induced root hair defect and the TOR network, which is likely to be affected due to the reduced availability of His. Cell wall integrity sensing mechanisms directly linked to the TOR kinase may be a plausible explanation for the observed suppression of the *LRX1ΔE14*-induced root hair defect by *sune82*, but the molecular details at a higher resolution on the subcellular level remain to be investigated.

## Supplementary data

The following supplementary data are available at *JXB* online.

Table S1. Primers used in this study.

Fig. S1. *sune82* plants display a dwarf phenotype.

Fig. S2. Expression pattern of *HISN2* and principle of the co-segregation analysis.

Fig. S3. Histidine supplementation can complement the *LRX1ΔE14 sune82* root phenotype.

Fig. S4. Homozygous *hisn2*^*crispr*^ mutants are lethal.

Fig. S5. The *sune82* mutation reduces sensitivity to the TOR inhibitor AZD-8055 and results in a shorter meristem.

Fig. S6. *sune106* and *ipms1* crispr mutations are in the first exon of *IPMS1*.

Dataset S1. Raw data of the results presented.

Dataset S2. Free amino acid quantification.

erae479_suppl_Supplementary_Dataset_S2

erae479_suppl_Supplementary_Table_S1_Figures_S1-S6_Dataset_S1

## Data Availability

All data can be found in Supplementary Dataset S1.

## Abbreviations:

EMS: ethyl methanesulfonate
FER: FERONIA
HISN2: histidine biosynthesis 2
IPMS1: isopropylmalate synthase 1
LRX: leucine-rich repeat extensin
MS: Murashige and Skoog
PRA-CH: phosphoribosyl-AMP cyclohydrolase
PRA-PH: phosphoribosyl-ATP pyrophosphatase
PI: propidium iodide
RALF: rapid alkalinization factor
RPS6: ribosomal protein S6
*sune*: *suppressor of dominant-negative LRX1ΔE14*
TOR: target of rapamycin.
