# Supplementary material for: Histidine limitation alters plant development and influences the TOR network
Source: J Exp Bot. 2024 Dec 17;76(4):1085–98. doi: 10.1093/jxb/erae479 (PMC11850971; doi:10.1093/jxb/erae479)
Supplement: erae479_suppl_Supplementary_Dataset_S2 [file erae479_suppl_supplementary_dataset_s2.pdf]

The concentration of 20 amino acids in all samples. The concentration was shown in μM.

Amino Acid	col_1	col_2	col_2_re	col_3	*sune82* _1	*sune82* _2	*sune82* _3	*sune82* _3 _re	*hisn2* _1	*hisn2* _1_re	*hisn2* _2	*hisn2* _3
Histidine	21.33	14.44	15.78	19.11	3.11	4.67	3.78	3.56	2.44	2.44	2.00	2.44
Asparagine	499.56	286.89	280.89	394.89	644.22	970.22	791.56	800.67	437.78	441.11	293.78	382.44
Serine	101.78	47.78	47.33	67.11	134.44	161.78	124.89	125.78	128.67	127.11	53.56	104.44
Glutamine	2892.22	1878.00	1878.44	2690.89	4208.67	6458.67	5437.78	5382.67	2860.22	2846.89	2146.89	2636.67
Arginine	168.00	115.78	121.33	186.44	249.11	340.44	323.78	308.22	154.00	151.78	117.33	138.00
Glycine	75.11	64.22	65.11	46.67	83.33	102.22	76.00	73.78	61.78	62.22	30.00	28.67
Aspartic acid	181.56	100.44	102.44	139.33	138.00	234.00	188.89	185.78	94.44	93.56	66.44	89.78
Glutamic acid	362.67	178.00	180.89	242.67	292.89	439.33	363.33	352.00	196.89	196.44	113.78	169.33
Threonine	76.44	36.89	36.44	54.44	70.22	99.11	82.67	83.56	58.22	59.56	34.44	51.11
Alanine	139.11	60.44	60.67	81.33	99.56	125.78	109.33	109.11	66.89	67.33	36.22	56.00
Proline	52.44	24.22	24.22	49.78	48.22	64.44	60.44	61.11	34.89	34.67	21.56	37.56
cystine	0.22	n.d.	n.d.	n.d.	n.d.	n.d.	n.d.	n.d.	n.d.	n.d.	n.d.	n.d.
Lysine	7.56	4.44	4.22	5.11	5.78	8.44	6.89	6.67	3.11	3.11	2.22	3.11
Tyrosine	2.22	0.89	0.89	1.33	1.56	1.56	1.33	1.33	0.89	0.89	0.44	0.67
Methionine	2.89	1.33	1.33	2.00	2.22	3.78	3.11	3.11	1.56	1.56	0.67	1.11
Valine	23.33	11.78	12.00	16.89	18.44	26.89	22.22	22.22	12.44	12.67	7.56	12.00
Isoleucine	5.78	2.89	2.89	4.00	4.00	5.56	4.22	4.44	2.67	2.67	1.56	2.44
Leucine	7.56	3.78	3.78	5.11	5.33	6.67	5.56	5.33	3.33	3.11	2.00	3.11
Phenylalanine	4.89	2.22	2.00	3.33	3.56	4.44	3.56	4.00	2.44	2.44	1.56	2.00
Tryptophan	n.d.	n.d.	n.d.	n.d.	n.d.	n.d.	n.d.	n.d.	n.d.	n.d.	n.d.	n.d.

Amino Acid	Mean Col	SD Col	Mean *sune82*	SD *sune82*	Mean *hisn2 crispr*	SD *hisn2 crispr*
Histidine	18.30	3.52	3.85	0.78	2.30	0.26
Asparagine	393.78	106.34	802.00	163.25	371.33	72.64
Serine	72.22	27.36	140.37	19.15	95.56	38.34
Glutamine	2487.04	536.96	5368.37	1126.60	2547.93	364.85
Arginine	156.74	36.65	304.44	48.64	136.44	18.38
Glycine	62.00	14.35	87.19	13.53	40.15	18.74
Aspartic acid	140.44	40.57	186.96	48.03	83.56	15.00
Glutamic acid	261.11	93.70	365.19	73.24	160.00	42.33
Threonine	55.93	19.82	84.00	14.49	47.93	12.20
Alanine	93.63	40.75	111.56	13.25	53.04	15.55
Proline	42.15	15.58	57.70	8.45	31.33	8.57
cystine	n.d.	n.d.	n.d.	n.d.	n.d.	n.d.
Lysine	5.70	1.64	7.04	1.34	2.81	0.51
Tyrosine	1.48	0.68	1.48	0.13	0.67	0.22
Methionine	2.07	0.78	3.04	0.78	1.11	0.44
Valine	17.33	5.79	22.52	4.23	10.67	2.70
Isoleucine	4.22	1.46	4.59	0.84	2.22	0.59
Leucine	5.48	1.92	5.85	0.71	2.81	0.71
Phenylalanine	3.48	1.34	3.85	0.51	2.00	0.44
Tryptophan	n.d.	n.d.	n.d.	n.d.	n.d.	n.d.
